# Topological guided-mode resonances: basic theory, experiments, and applications

**DOI:** 10.1515/nanoph-2024-0612

**Published:** 2025-03-07

**Authors:** Yu Sung Choi, Chan Young Park, Soo-Chan An, Jung Hyeon Pyo, Jae Woong Yoon

**Affiliations:** Department of Physics, 26716 Hanyang University, Seoul, 133-791, Korea

**Keywords:** guided mode resonances, topological physics, diffraction grating, waveguide, non-Hermitian Hamiltonians

## Abstract

Guided-mode resonance (GMR) is a key principle for various nanophotonic elements in practice. In parallel, GMR structures offer an efficient experimental platform for fundamental study of novel wave phenomena because of its versatile capability to synthesize complicated potential distributions and analyze deep internal properties conveniently in the optical far-fields. In this paper, we provide a brief review of topological GMR effects as a promising subtopic of the emerging topological photonics. Starting from a conceptually minimal model, we explain basic topological parameters, associated optical properties, experimental realizations, and potential applications. We treat topics of recent interest including topological edge-state resonances, deterministic beam shaping and mode matching, bound states in the continuum, unidirectional resonances, and polarization vortices. We finally address limitations, remaining challenges, and perspective of the topic.

## 1 Introduction

The discovery of topological insulators revealed the existence of robust electronic states protected by topological invariants [1], [2], [3], [4], [5], [6], [7]. This concept has since inspired topological photonics that has proposed new ways of robust light control with additional degrees of freedom associated with topological phases of electromagnetic structures and fields [8], [9], [10], [11], [12], [13], [14], [15], [16], [17], [18], [19], [20], [21]. Intriguingly, photonics has substantially enriched the topological physics with its unparalleled experimental feasibility to create sophisticated potential distributions for fundamental study [22], [23], [24], [25], [26], [27], [28], [29], [30], [31], [32], [33] as well as abundant application areas including signal processing, telecommunications, display, rapid nondestructive sensing and detection, quantum information technology, and many others [34], [35], [36], [37].

Along this line, guided-mode resonance (GMR) structures [38], [39], [40] have emerged as a promising platform because of their relative simplicity in structure designs, versatile spectral properties, convenience in measurement and analyses through inherently coupled far-field radiation channels, and immediate application potential to nanophotonic component engineering [41], [42], [43], [44], [45], [46], [47], [48], [49], [50], [51], [52].

In this paper, we provide a review of topological GMR effects and their state of the art. We explain a minimal model that describes guided mode and far-field properties in terms of band topology of 1D or 2D topological insulators and Dirac fermions. The model explicitly reveals relations between fundamental design parameters and topological GMR effects such as band inversion, bound state in the continuum (BIC), non-Hermitian modal degeneration and singularity, localization at edges and defects, and broadband unidirectionality. We highlight experimental demonstrations that validate these theoretical concepts and showcase their practical applications. Finally, we address present limitations and challenges to provide a proper perspective.

## 2 Minimal model of guided-mode resonances

The theoretical foundation of topological GMR effects is based on the coupled-mode theory developed by Kazarinov and Henry in 1985 [53]. Although this approach was originally proposed to describe second-order distributed feedback lasers, it provides a conceptually simplest but powerful analytical tool for describing various GMR effects in its most useful spectral domain around the second-order Bragg condition, where resonant excitation and coupled radiation efficiencies are maximal in general.

**Figure 1:**
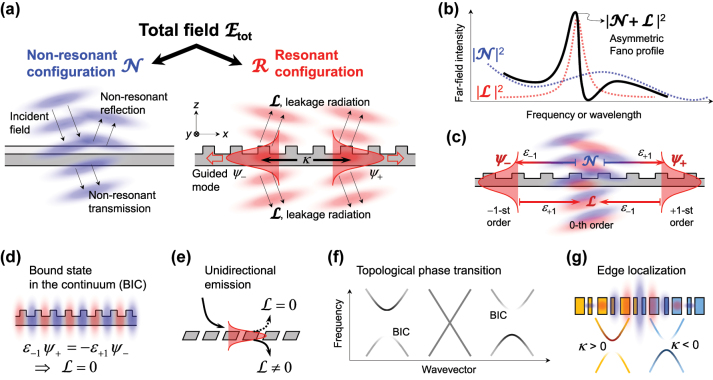
A minimal model of guided mode resonances (GMRs). (a) Total field ℰ_tot_ decomposition into the non-resonant N and resonant ℛ configurations. N is a solution for a homogeneous-effective-medium multilayer structure while ℛ consists of a linear superposition of guided modes *ψ*_±_ and leakage radiation ℒ. (b) Characteristic Fano profile of a GMR as a result of configuration interference between spectrally broad N feature and narrow Lorentzian ℛ ≈ ℒ feature in the optical far field. (c) Basic diffractive coupling pathways between N , *ψ*_±_, and ℒ. *ε*_*m*_ denotes the *m*-th Fourier harmonic amplitude of the dielectric function in the grating layer. (d–g) Examples of various GMR phenomena: (d) Bound state in the continuum. (e) Unidirectional coupling and radiation in vertically asymmetric gratings. (f) Topological phase transition in spectral band structures. (g) Edge-localized GMR at a topological junction.

This model starts with an ansatz for total field ℰ_tot_ as

(1) $$E_{tot}=\left[N\left(z\right)+R\left(x,z\right)\right]e^{ikx},$$

(2) $$R\left(x,z\right)=\left(\psi_{+}e^{+iqx}+\psi_{-}e^{-iqx}\right)u\left(z\right)+L\left(z\right)$$

for given Bloch wave vector *k*, grating vector *q*, and normalized cross-sectional wave function *u*(*z*) of the guided mode. These field expressions are schematically illustrated in Figure 1a. ℰ_tot_ is linearly decomposed into non-resonant configuration N and resonant configuration ℛ. The non-resonant configuration N is a solution to the electromagnetic wave equation for a simple multilayer system where the grating layer is replaced by an effective homogeneous medium. Therefore, N is simply a linear superposition of incident, non-resonantly reflected, non-resonantly transmitted, and intra-layer planewave fields. N is completely predetermined by using the Fresnel equations for given incident field in advance of complete description of ℰ_tot_.

ℛ denotes the resonant configuration which is a linear superposition of guided mode fields *ψ*_±_ and their leakage radiation field ℒ, as indicated in Eq. (2). Therein, *ψ*_±_ describes the ±1-st-order harmonic fields while ℒ separately describes the 0-th-order harmonic field. Subsequently, the 0-th-order of the total field is described by the linear superposition N + ℒ that covers the entire space and the ±1-st-order harmonic fields are denoted by another linear combination *ψ*_+_ *e* ^+*iqx*^ + *ψ*_−_ *e* ^−*iqx*^ that is localized at the waveguide grating with the guided-mode wavefunction *u*(*z*).

Keeping these field-decomposition configurations in mind, one can intuitively describe the effect of the grating. The diffraction grating leads to resonant N -ℛ coupling and subsequent excitation of *ψ*_±_ and ℒ. We note that the existence of ℒ makes the guided-mode *ψ*_±_ system a non-Hermitian eigen-system in general. Such coupling and resonant excitation is properly described by plugging the total-field ansatz in the electromagnetic wave equation

(3) $$\left[\frac{\partial^{2}}{{\partial x}^{2}}+\frac{\partial^{2}}{{\partial x}^{2}}+\frac{\omega^{2}}{c^{2}}\varepsilon \left(x,z\right)\right]E_{tot}=0.$$

Equation (3) yields closed-form solutions for resonant field ℛ consisting of guided-mode amplitudes |*ψ*⟩ = [*ψ*_+_ *ψ*_−_]^T^ and leakage-radiation wave function ℒ as

(4) $$\left|\psi \right\rangle =\omega_{0}{\left(H_{\text{GMR}}-\Delta \omega \right)}^{-1}\left|D\right\rangle ,$$

(5) $$L\left(z\right)=\left(\varepsilon_{-1}\psi_{+}+\varepsilon_{+1}\psi_{-}\right)w\left(z\right).$$

Here, ℋ_GMR_ is a 2 × 2 matrix effective Hamiltonian that we will explain in detail later in this section, *ω*_0_ is frequency of the second-order Bragg condition for the guided mode, and Δ*ω* = *ω* − *ω*_0_ is frequency detuning. Diffractive N -to-ℛ coupling amplitudes |*D*⟩ = [*D*_+1_ *D*_−1_]^T^ and leakage-radiation wave function *w*(*z*) are, respectively, given by the following relations.

(6) $$\left|D\right\rangle =\left[\begin{array}{c} \varepsilon_{+1}\\ \varepsilon_{-1} \end{array}\right]\frac{n_{\text{p}}}{2n_{\text{g}}}\overset{+\infty }{\underset{-\infty }{\int }}dz\;u^{*}\left(z\right)N\left(z\right),$$

(7) $$w\left(z\right)=-\frac{\omega_{0}^{2}}{c^{2}}\underset{\text{Grating}\;\text{layer}}{\int }dz^{\prime }\;G\left(z,z^{\prime }\right)u\left(z^{\prime }\right),$$

where *ε*_*m*_ is *m*-th harmonic Fourier amplitude of the dielectric function in the grating layer and *G*(*z*,*z*′) is 1D Green’s function for the effective homogeneous film structure that leads to the non-resonant field solution N .

Equations (4)–(7) are basically approximate solutions because the field ansatz ruled out high-order harmonic fields beyond the ±1-st orders and we take first-order perturbation for all inter-modal diffractive coupling events. Nevertheless, they capture dominant underlying physics in remarkably intuitive ways at the expense of losing quantitative accuracy.

First, Eqs. (4) and (6) describe the guided-mode amplitudes |*ψ*⟩ and leakage radiation ℒ as creating a standard Lorentzian resonance with the factor (ℋ_GMR_ − Δ*ω*)^−1^, where Re(ℋ_GMR_) and Im(ℋ_GMR_) denote the resonance center frequency and decay rate, respectively.

In the optical far field, the resonant field ℛ tends to leakage-radiation field ℒ and subsequently the total field ℰ_tot_ is described by

(8) $$E_{tot}\approx \left[N\left(z\right)+L\left(z\right)\right]e^{ikx}.$$

Therefore, interference between N as a slowly-varying spectral background and ℒ as a narrow Lorentzian resonance line naturally leads to asymmetric spectral line shape in the optical far field. See Figure 1b for the schematic diagram for this point. We note that this interaction picture is fully consistent with canonical Fano resonance as resulting from the configuration interference between the resonant and non-resonant transition pathways in general [54]. Therefore, the key features of Eqs. (4)– (7) are applicable to resonant scattering responses of 2D photonic-crystal slabs [55].

Causal coupling processes are also properly and intuitively described within this approach. According to Eqs. (4) and (6), the guided-mode amplitude *ψ*_±_ is explicitly driven by the ±1-st order diffraction (same sign in the same order) of the non-resonant field N , respectively, and their magnitude scales with space overlap of the guided-mode wave function *u*(*z*) and the non-resonant field N . The same description applies to the leakage-radiation field ℒ in Eq. (5) combined with Eq. (7). Looking at Eq. (7), the normalized leakage-radiation wave function *w*(*z*) is simply a collection of secondary waves *G*(*z*,*z*′) scattered from the normalized guided mode *u*(*z*). Equation (5) describes ℒ as created by the ∓1-st order diffraction from *ψ*_±_ (opposite sign in the same order), which is consistent with our standard intuition. These coupling processes are graphically summarized in Figure 1c.

This model can be used to describe leaky guided-mode eigen system. The leaky eigen system is derived from Eq. (4) for the self-oscillating case in the absence of the incident field. In such a condition where N = 0 and |*D*⟩ = 0, nontrivial solution for |*ψ*⟩ is possible only if the Lorentzian factor (ℋ_GMR_ − Δ*ω*)^−1^ is singular. Therefore, Eq. (4) reduces to an eigenvalue problem as

(9) $$H_{\text{GMR}}\left|\phi \right\rangle =\Delta \omega_{\phi }\left|\phi \right\rangle .$$

The leakage radiation ℒ_*ϕ*_ for the guided-mode eigen state is simply determined by taking |*ψ*⟩ = |*ϕ*⟩ = [*ϕ*_+_ *ϕ*_−_]^T^ in Eq. (5). Equation (9) allows us a comprehensive leaky-mode band analysis including frequency band structures and their linewidth distributions in connection with modal-field patterns and leakage-radiation characteristics, which constitute the essence of various phenomena including regular GMRs, bound states in the continuum (BIC), radiation unidirectionality, topological phase transition and localization, as schematically illustrated in Figure 1d–g.

## 3 Basic topological parameters

The eigenvalue problem in Eq. (9) connects GMR effects with 1D Dirac fermions or basic 1D topological insulators. ℋ_GMR_ can be viewed by a simple unitary transformation of 1D Dirac Hamiltonian ℋ_Dirac_, which tight-binding Hamiltonian ℋ_SSH_ of a dimerized atomic-chain lattice, also known as Su–Schrieffer–Heeger (SSH) model, reduces to in the low-energy continuum approximation [56], [57], [58]. In our GMR coupled-mode model, ℋ_GMR_ in general takes a particular expression as

(10) $$H_{\text{GMR}}=kv_{\text{g}}\sigma_{3}+\kappa \sigma_{1}=\left[\begin{array}{cc} kv_{\text{g}} & \kappa \\ \kappa & -kv_{\text{g}} \end{array}\right],$$

where *v*_g_ is group speed of the guided mode, **σ**_*j*_ is the Pauli matrix, and *κ* is coupling rate between the right-going (*ψ*_+_) and left-going (*ψ*_−_) guided modes or equivalently the second-order Bragg-reflection rate of the guided mode [59]. One can relate the 1D Dirac Hamiltonian

(11) $$H_{\text{Dirac}}=mc^{2}\sigma_{3}-pc\;\sigma_{1}=\left[\begin{array}{cc} mc^{2} & -pc\\ -pc & -mc^{2} \end{array}\right]$$

through a unitary transformation such that

(12) $$UH_{\text{Dirac}}U^{†}=\hbar \;H_{\text{GMR}},$$

(13) $$U=\frac{1}{\sqrt{2}}\left(\sigma_{0}-i\sigma_{2}\right)=\frac{1}{\sqrt{2}}\left[\begin{array}{cc} 1 & -1\\ 1 & 1 \end{array}\right].$$

Here, we assume the following parametric correspondence

(14) $$p=\hbar k,c=v_{g},\;\text{and}\;mc^{2}=\hbar \kappa .$$

This parametric correspondence makes good physical sense with no need of introducing any additional, contrived assumptions. Therefore, an identical underlying physics applies to GMRs, 1D Dirac fermions, and 1D topological insulators (SSH model) as far as we concern their wave-kinematic properties despite their fundamental differences.

The correspondence in Eqs. (12)– (14) implies the inter-guided-mode coupling constant *κ* as a key topological parameter in analogy to mass *m* in the Dirac equation. The sign of *κ* determines topological phase of a given GMR system, *i.e.*, *κ* > 0 for trivial phase and *κ* < 0 for topological phase. Transition of the system between these two phases accompanies nonadiabatic changes in the band structure and spatial symmetry of eigenstates. As *κ* changes its sign, the photonic bandgap closes and reopens with a linear band crossing at *κ* = 0 in analogy to a Dirac point in electronic systems. In the trivial phase (*κ* > 0), the lower band edge corresponds to a sub-radiant state (often referred to as a bound state in the continuum, BIC) with odd symmetry in the in-plane wave function while the upper band edge is a super-radiant state with even symmetry. This order is reversed in the topological phase (*κ* < 0).

Although the sign of *κ* provides a straightforward way to distinguish between topological phases, more sophisticated characterizations offer deeper insights into the topological nature of GMR systems. One example is polarization vortices around specific wavevector-space points or lines associated with a BIC [60], [61], [62], [63], [64]. The leakage radiation field ℒ carries crucial information about the topological properties through its polarization state. The radiation polarization state of each resonant mode can be mapped onto the momentum space [65] such that **L**(**k**) = ℒ_*x*_(**k**)**e**_*x*_ + ℒ_*y*_(**k**)**e**_*y*_, where **k *=*** (*k*_*x*_, *k*_*y*_) is in-plane wavevector and the components ℒ_*x*_(**k**) and ℒ_*y*_(**k**) are determined by *w*(*z*) from Eq. (7) [66], [67], [68], [69]. The BICs correspond to the intersections of the nodal lines of ℒ_*x*_(**k**) = 0 or ℒ_*y*_(**k**) = 0 on *k*_*x*_-*k*_*y*_ plane. The polarization-vortex charge *q* is thus defined as

(15) $$q=\frac{1}{2\pi }\oint_{C}dk\cdot \nabla_{k}\varphi \left(k\right),$$

where *φ*(**k**) = arg[ℒ_*x*_(**k**)+*i*ℒ_*y*_(**k**)]. Non-trivial *q* is naturally obtained for any contour *C* enclosing a singular point for **L**(**k**) = 0. At such points, the decoupling of guided modes *ψ*_±_ from the radiation channels satisfies specific conditions that can be driven from Eqs. (4) and (5).

Another important topological invariant in GMR systems is Zak phase *γ* [70]. It is a Berry phase acquired by the Bloch states as they evolve across the Brillouin zone and thereby defined by

(16) $$\gamma =\oint_{1\text{st}\;\text{BZ}}i\left\langle u_{k}\right|\nabla_{k}\left|u_{k}\right\rangle dk,$$

where |*u*_*k*_⟩ is the periodic part of the eigenstate wave function. *γ* provides a global characterization of the band topology and is particularly important when one investigates localized states at interfaces between topologically distinguished regions.

The topological invariants *q* and *γ* offer a remarkably straightforward approach to understanding properties of various GMR phenomena such as BICs [71], unidirectional resonances [66], and lateral localization at the grating boundaries and interfaces [72], [73]. These invariants not only characterize the topological nature but also provide powerful tools for manipulating light in GMR structures, as we will explore in the following sections.

## 4 Edge-state guided-mode resonances

### 4.1 Theoretical foundations and basic properties

The Jackiw-Rebbi (JR) solution as a topological edge state was originally developed in the context of quantum field theory [58] and exactly the identical concept applies to GMR systems at certain conditions. In the original context, a JR solution is a zero-energy eigenstate of the 1D Dirac equation for a domain-wall problem where *m*(*x*) distribution contains a junction (*x* = *x*_0_) between two topologically distinguished regions, *i*.*e*., *m*(*x* < *x*_0_) < 0 for the topological-phase region and *m*(*x* > *x*_0_) > 0 for the trivial-phase region. A detailed expression for JR state |JR⟩_Dirac_ is

(17) $${\left|\text{JR}\right\rangle }_{\text{Dirac}}=\frac{1}{\sqrt{2}}\left[\begin{array}{c} 1\\ i \end{array}\right]f\left(x\right),$$

(18) $$f\left(x\right)=\exp\left[-\frac{c}{\hbar }\overset{x}{\underset{x_{0}}{\int }}m\left(x^{\prime }\right)dx^{\prime }\right].$$

Here, *f*(*x*) is an envelope function that describes the localization profile. Equation (17) describes the localized domain-wall state at a topological junction for given Dirac-mass distribution *m*(*x*).

Corresponding guided-mode state to the JR solution in Eqs. (17) and (18) are found by taking parametric correspondences in Eq. (14) and a unitary transformation as

(19) $${\left|\text{JR}\right\rangle }_{\text{GMR}}=U{\left|\text{JR}\right\rangle }_{\text{Dirac}}=\frac{1}{\sqrt{2}}\left[\begin{array}{c} e^{-i\pi /4}\\ e^{+i\pi /4} \end{array}\right]f\left(x\right),$$

(20) $$f\left(x\right)=\exp\left[-\frac{1}{c}\overset{x}{\underset{x_{0}}{\int }}\kappa \left(x^{\prime }\right)dx^{\prime }\right],$$

where *κ*(*x*) is now a position-dependent coupling constant. From this exact analogy, the JR state represents a localized guided mode at the interface between two regions in the trivial and topological phases.

In conventional GMR systems, the non-local nature of guided modes leads to unfavorable degradation of key performance metrics such as resonance quality (Q) factor as lateral footprint width of structure decreases below effective propagation distance of the guided mode [74], [75], [76], [77]. The GMR JR state can be employed to avoid this problem with no need of supplement structures such as side reflectors [78], [79], [80], [81], [82], [83].

**Figure 2:**
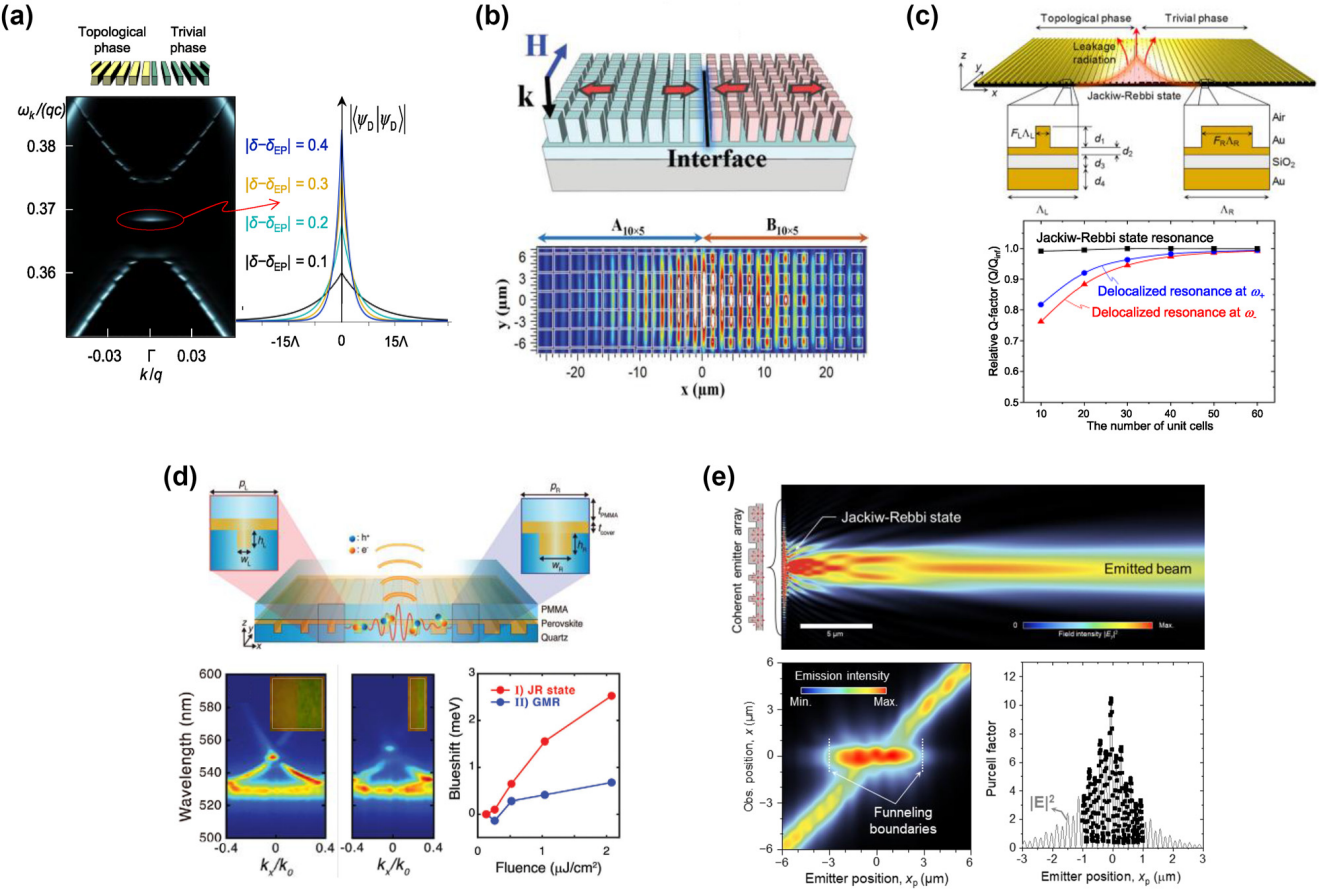
Photonic Jackiw–Rebbi states. (a) Spectral feature and lateral confinement of JR state. (b) 2D resonant topological edge states. (c) Topological surface plasmon resonance and size-independent feature. (d) Topological exciton polaritons in a compact junction and large nonlinear blueshift. (e) Topological beaming of light, funneling and Purcell enhancement. (a) Reproduced with permission [59]. Copyright 2021, De Gruyter. (b) Reproduced with permission [84]. Copyright 2022, Wiley-VCH. (c) Reproduced with permission [85]. Copyright 2023, Elsevier B.V. (d) Reproduced with permission [86]. Copyright 2024, Wiley-VCH. (e) Reproduced with permission [87]. Copyright 2022, AAAS.

The first attempt is discussed in Ref. [59], as shown in Figure 2a. The GMR JR state shows the characteristic bi-exponential decaying profile in consistency with Eq. (20) and tightly localized to the interface between two topologically distinguished gratings. In this numerical demonstration, only 30 periods are required to fully accommodate this GMR state. 2D extension of this type of compact GMR structure was proposed in [84], as shown in as shown in Figure 2b.

The GMR JR state has been utilized to enhance various nanophotonic properties associated with surface plasmon polaritons and exciton polaritons. A surface plasmon-polariton JR state in deep-subwavelength metal gap was demonstrated in Ref. [85], Figure 2c. They designed a metal-insulator-metal (MIM) zero-order grating structure to create such state and remarkable improvement in the optical confinement from the all-dielectric counterpart was numerically demonstrated.

Exciton-polariton JR-state resonance was demonstrated in [86], Figure 2d. The exciton-polariton JR-state resonance was experimentally realized in perovskite waveguide grating structures. They showed remarkable enhancement of exciton-polariton spontaneous emission and nonlinear blue shift which encourages follow-up study for efficient surface-emitting polaritonic lasers.

Although these GMR edge states have provided a fertile ground for both fundamental studies and practical implementations [88], [89], [90], it is of great interest to extend the topic to other topological boundary states, which have been researched to other photonic systems, such as corner states [91], [92], [93], [94], hinge states [95], [96], and other higher-order topological states [97], [98]. These boundary states have been extensively explored across different photonic platforms, leading to fundamental discoveries and practical applications in photonic crystals, metamaterials, and coupled resonator arrays.

The inherent non-Hermitian nature of GMR states gives rise to unique properties particularly in their interaction with free-space radiation. GMR systems inherently couple to the radiation continuum in the form of guided-mode’s leakage radiation ℒ. In topological GMR structures, ℒ can be efficiently controlled by manipulating topological parameters including Dirac mass *m* or equivalently second-order Bragg reflection rate constant *κ*. Interplay between the topological parameters and radiation continuum also leads to an anomalous beam-shaping effect associated with exceptional points [99].

Topological ℒ control was first demonstrated by Lee et al. [87], Figure 2e. They demonstrated emission of a well-defined beam from a point source at a topological GMR-grating junction. Their analysis revealed an electromagnetic funneling effect due to particular field patterns implied in the GMR JR state. This effect highlights a unique ability of topological GMR systems to control light propagation and emission in a remarkably simple structure. Furthermore, experimental validation of these properties was provided by An et al. [86]. They observed an exciton-polaritonic beam emission by mediation of the exciton-polariton JR state that couples to ℒ within a narrow angle predicted by the theory.

### 4.2 Deterministic beam shaping

Topological beam shaping is an approach that leverages the unique properties of topological GMRs to control leakage radiation ℒ. This technique builds upon the photonic JR states discussed in the previous section. According to Eq. (20), JR state envelope *f*(*x*) and second-order Bragg-reflection rate constant *κ*(*x*) are locally related by

(21) $$\kappa \left(x\right)=-\frac{1}{f}\frac{df}{dx}.$$

Therefore, one can obtain any desired *f*(*x*) by systematically encoding it into *κ*(*x*) following Eq. (21). Since the leakage radiation ℒ is nothing but the first-order diffraction field from the JR state according to Eq. (5), lateral profile of ℒ primarily takes *f*(*x*) profile with the fast oscillation by *e* ^±*iqx*^ being completely removed from its in-plane distribution. Thereby we obtain ℒ ≈ *f*(*x*)⋅*constant*. The required *κ*(*x*) distribution can be readily obtained by appropriate unit-cell designs that control *ε*_± 1_^2^ and *ε*_± 2_ as primary factors determining the second-order Bragg reflection strength and phase.

**Figure 3:**
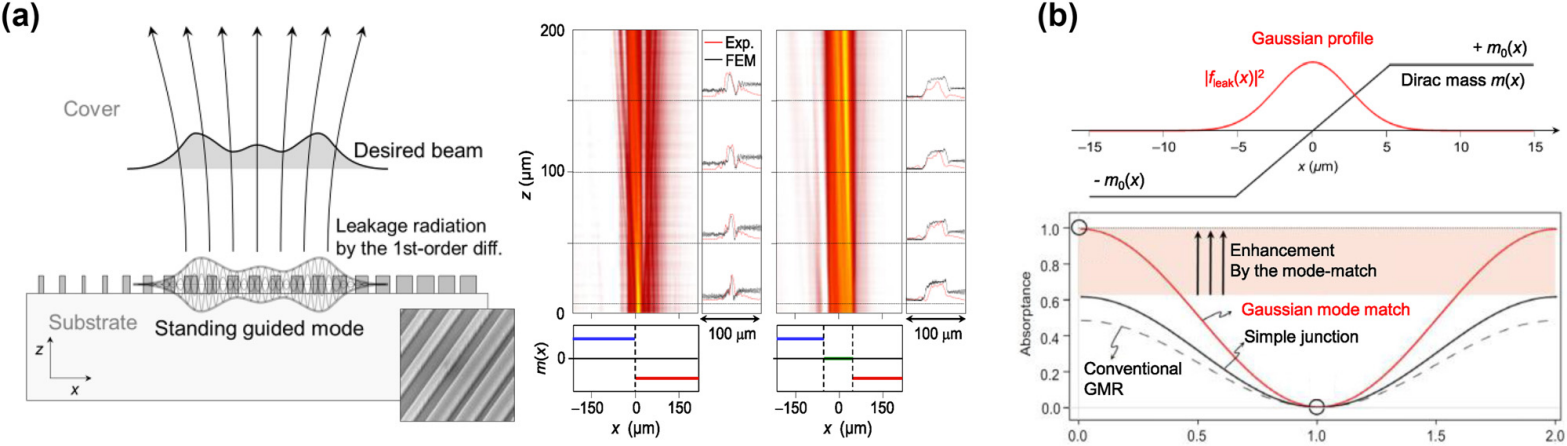
Topological beam shaping via mass engineering. (a) Experimental demonstration of topological beam shaping. (b) Coherent perfect absorbers based on topological mode-matching to a Gaussian beam. (a) Reproduced with permission [101]. Copyright 2024, ArXiv. (b) Reproduced with permission [102]. Copyright 2024, Nature Publishing Group.

This confinement and beam shaping approach offer advantages over conventional GMR systems in consideration of a lateral mode-matching problem with non-planewave incidence in practice [75]. Apodization and trial-solution-based aperiodic structure optimization have been used for solving the mode-matching problem [99], [100]. These approaches often require complex algorithms with extremely high number-crunching power and do not guarantee a proper solution. In contrast, Eq. (21) provides a direct, forward design rule for shaping ℒ whose time reversal is exactly the ideally optimal beam that most efficiently excites the resonance. Demonstration of this new approach is found in [87], [101]. Therein, they experimentally realized a flat-top beam profile, as shown in Figure 3a. In [102], the same approach was used to obtain a Gaussian beam in order to maximize efficiency of GMR coherent absorbers, as shown in Figure 3b.

Despite these advancements, challenges remain in expanding the degrees of freedom in *κ* control, particularly in single-part unit-cell structures where fill factor of the grating ridge serves as the only tuning parameter. In [103], a bilayer configuration was proposed within this context. It introduces a lateral shift between the two layers as an additional parameter that efficiently control *κ*.

## 5 Polarization vortices

**Figure 4:**
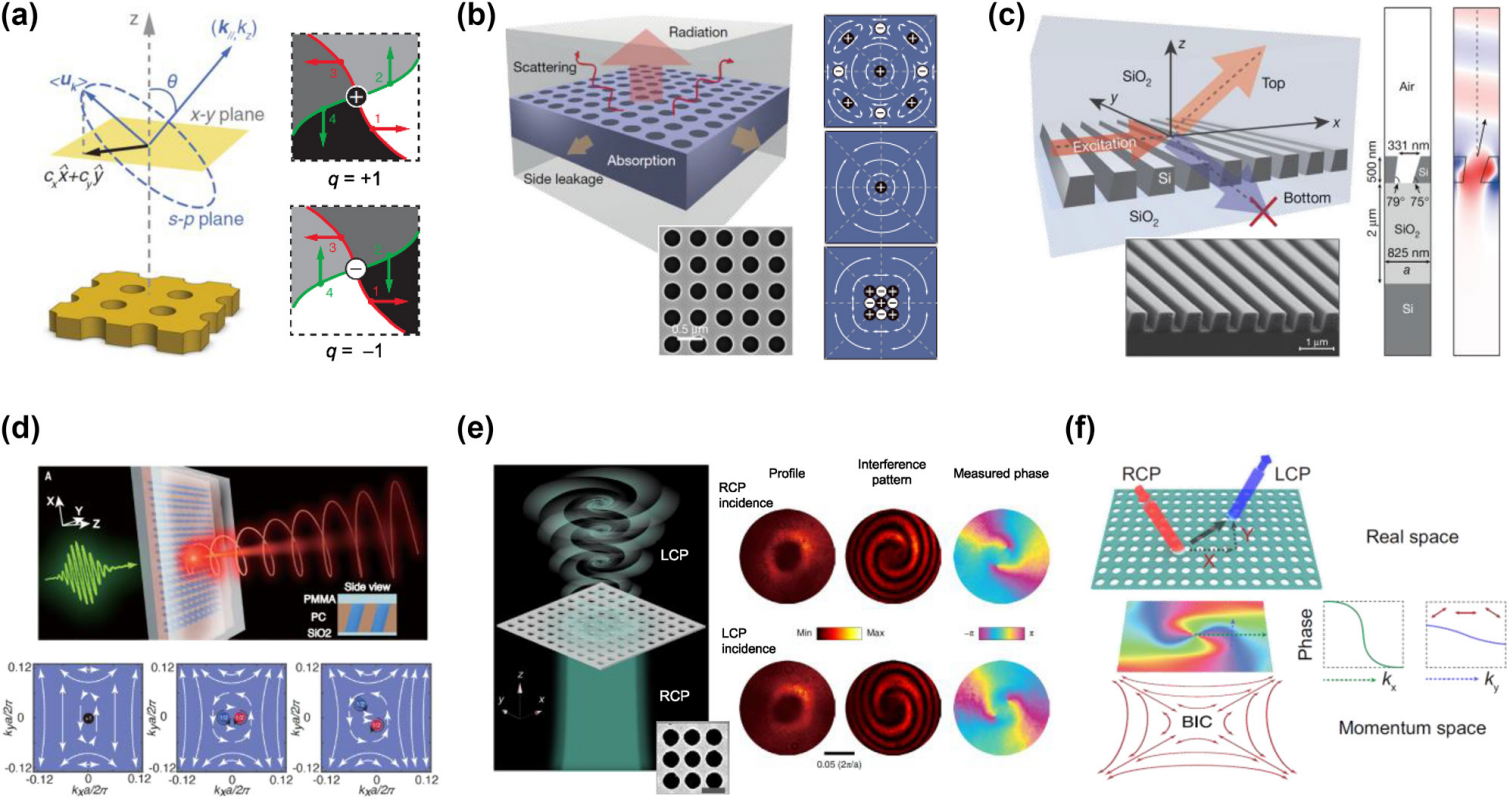
Topological nature of bound state in the continuum in guided mode resonances. (a) Momentum-space polarization distribution in a photonic crystal slab, illustrating the topological vortex structure around a BIC. (b) Ultra high-Q resonances by merging multiple topological charges. (c) Topologically enabled unidirectional GMRs. (d) Chiral emission from quasi-BIC. (e) Optical vortex beam around BICs. (f) Spin hall effect of light via topological vortices. (a) Reproduced with permission [65]. Copyright 2014, American Physical Society. (b) Reproduced with permission [105]. Copyright 2019, Nature Publishing Group. (c) Reproduced with permission [66]. Copyright 2020, Nature Publishing Group. (d) Reproduced with permission [106]. Copyright 2022, AAAS. (e) Reproduced with permission [107]. Copyright 2020, Nature Publishing Group. (f) Reproduced with permission [108]. Copyright 2022, American Physical Society.

The resonant configuration ℛ in GMR involves unique topological features in its far-field polarization characteristics. These features arise naturally from our minimal model described in Section 2. The leakage radiation field ℒ in Eqs. (5) and (7) become singular at specific points in momentum space where its polarization becomes undefined as shown in Figure 4a [65]. These singularities arise from the specific coupling conditions between guided modes and their radiation channels, as described by Eqs. (4) and (5). The polarization vectors of radiation fields reveal that a BIC produces a polarization singularity in momentum space due to radiation cancellation. Therefore, a BIC is a sort of singularity at which polarization direction of ℒ cannot be uniquely defined and thereby the only physically legitimate case is ℒ = 0 [71], [104]. In the theory in Section 2, the decoupling of ℛ with the radiation continuum is understood in Eq. (5) by complete destructive interference between contributions from *ψ*_±_ in ℒ due to symmetry mismatch (symmetry-protected BICs) or by continuous parameter tuning (accidental BICs).

The polarization topology can be quantified with polarization-vortex charge *q* defined in Eq. (15). In this approach, a BIC is treated as a topological defect carrying *q* in a GMR system. The polarization-vortex charges are conserved quantities as far as they do not move outside the light cone nor encounter other vortex charges [65]. The distribution of BICs in momentum space follows the space group symmetry of photonic crystal slabs. Direct observation of polarization vortices near the mode center has validated this interpretation [109], [110], [111]. This understanding has led to advances in parametric BIC control [67], [69], [71], [104], [112], vortex beam generation [107], [113], [114], [115], [116], [117], [118], [119], [120], [121], unidirectional radiation [66], [122], [123], [124], [125], [126], [127], [128], [129], [130], and chiral BICs [106], [131], [132], [133], [134], [135], [136], [137], [138], [139], [140], [141], [142], [143].

Manipulation of polarization-vortex charges includes BIC merging processes [105], [144], [145], [146], [147], [148], [149], [150], [151]. An important demonstration is introduced in Figure 4b [105]. It treats a band containing a C_2_ symmetry-protected BIC at Γ point together with multiple accidental BICs away from Γ point. An isolated BIC with *q* = ±1 exhibits quality factor scaling of Q ∝ Δ*k* ^−2^, where Δ*k* represents the wavevector distance from the BIC point. When multiple BICs merge, this scaling law dramatically changes to Q ∝ Δ*k* ^−6^. Further studies have explored high-order charges merging at Γ point in high-symmetry systems [145], [150]. Additional research has investigated BIC merging phenomena at off-Γ points in systems with and without symmetry breaking perturbations [144], [147].

Symmetry-breaking perturbations provide a convenient way for manipulating polarization-vortex charges. At high symmetry points, BICs can carry higher vortex charges. When a higher symmetry is broken while preserving C_2_^z^ symmetry, the BIC splits into multiple BICs with lower charges [152]. If C_2_^z^ symmetry is broken, a BIC splits into pairs of quasi-BICs carrying half-integer charges with opposite chirality [153]. Conversely, C points with opposite signs at identical magnitudes can merge into a single BIC [154]. Furthermore, pairs of C points carrying opposite half charges and same chirality can be generated [122], [126]. Breaking up-down mirror symmetry induces vertically asymmetric radiation patterns. This asymmetry in the radiation fields enables unidirectional guided-resonance that selectively radiate in a single preferred direction, as demonstrated in Figure 4c [66].

Through continuous parameter tuning in such symmetry-broken structures, half-integer charges can recombine into an integral charge with uniquely different leakage-radiation properties. While realizing unidirectional GMR typically requires advanced fabrication techniques for asymmetric sidewalls [91], [92], [93], [94], [95], [96], an alternative approach using vertical-sidewall structures was demonstrated in [97]. This work achieved unidirectionality through hybrid guided modes, where inter-modal coupling between even-like and odd-like modes creates a field configuration with broken mirror symmetry.

These symmetry breaking effects further enable novel methods for chiral light-matter interactions. A particularly promising direction is chiral quasi-BICs operating at Γ point. When C_2_^z^ symmetry is broken, off-Γ C points emerge and induce extrinsic chirality under off-normal incidence [132], [134]. However, by carefully engineering up-down mirror symmetry perturbations, these C points can be deterministically tuned back to Γ point, resulting in quasi-BICs that combine strong chirality with remarkably high Q factors [106], [137]. This concept was demonstrated in GMR structures incorporating slanted sidewalls that simultaneously break both C_2_^z^ and vertical mirror symmetries. Operating at visible frequencies, these structures enable chiral emission with extremely high field enhancement while maintaining highly directional output with minimal angular divergence, as experimentally verified in Figure 4d [106].

By controlling incident light distribution, BICs enable rich physics in light-matter interactions through spin-orbit coupling. The vortex charges of BICs give rise to strong spin-dependent phenomena associated with geometric phase effects. Specifically, these vortices create spin-dependent lateral shifts through wavevector-dependent Pancharatnam-Berry (PB) phase gradients and cross-polarized resonant phase gradients. Such geometric phase effects can be harnessed to generate optical vortex beams and control the real-space position of light beams as shown in Figure 4e [107], and 4f [108], respectively. A manifestation of this effect is found in the spin-dependent far-field properties. When circularly polarized light interacts with strong polarization anisotropy near BICs, the transmitted cross-polarized radiation acquires a geometric phase. This results in the outgoing beam carrying orbital angular momentum *l* = ∓2*q* [107]. Such vortex charges are also known to induce a distinctive spin Hall effect characterized by spin-dependent lateral beam shifts in oblique planes [108].

Recent developments have shown remarkable progress in practical applications of topological GMR systems. For example, Zhang et al. [155] recently demonstrated that topological charges can be actively manipulated by integrating phase change materials into GMR structures. Metal-insulator phase transition of GeSbTe (GST) switches its refractive index between 4.724 and 5.96. Including this effect, they achieved continuous beam steering up to 160° with high radiation efficiency exceeding 80 %. This large-range tuning capability arises from the deterministic manipulation of half-integer charge merging and splitting.

The application scope of polarization vortices has also expanded significantly. Liu et al. [121] demonstrated that resonance-enabled topological darkness in photonic crystal slabs can be exploited to generate spatiotemporal optical vortices (STOVs). Through complete polarization conversion between circular polarizations, they successfully imprinted vortex singularities onto ultrashort reflected pulses and provided the first observation of such vortex lines using time-resolved spatial mapping. In related works, Xia et al. [156] demonstrated spin-locked vortex emission by integrating WS_2_ monolayers with photonic crystal slabs. By exploiting symmetry-protected BICs, they showed that GMRs with azimuthal polarization distribution can impose spin-correlated spiral phase fronts on the exciton emission. This achievement establishes a promising route toward valley-polarized structured light generation using two-dimensional materials.

These advances in polarization vortex control have enabled various functionalities beyond conventional optical vortex generation, from optical communications utilizing dynamically tunable vortex beams to LiDAR systems benefiting from wide-angle beam steering. While challenges remain for substantive practical implementations, alternative low-loss phase-change materials like Sb_2_Se_3_ and ongoing device optimization continue to push the boundaries of structured light manipulation capabilities.

## 6 Summary and perspective

We have reviewed fundamental theory and recent progress in topological GMR study. We have explained a minimal intuitive model that comprehensively captures essential underlying physics of resonant excitation, eigen-system formation and its connection to the radiation continuum, configuration interference with the incident and non-resonant fields, and subsequent spectral properties. On that basis, we briefly introduce various topological effects such as edge-state resonances, deterministic beam shaping and mode matching, bound states in the continuum, and unidirectional GMRs in their connection to basic topological parameters – Dirac mass, Zak phase, and polarization vortex charge.

These topological phenomena based on simple intuitive models have facilitated advanced applications. For example, merging polarization-vortex charges and topologically enhanced Goos-Hänchen shift have greatly improved refractometric sensitivity for biochemical sensor applications [157], [158]. In light source applications, both optically [159], [160] and electrically [161], [162] pumped vortex-beam lasers have been realized by integrating transition metal dichalcogenide (TMD) materials [156]. Dynamic topological charge control have been demonstrated by using phase-change perovskite materials [163] and GST-based structures [155]. In nonlinear applications, specially designed topological GMR structures can be used to significantly enhance the nonlinearity [67]. The development of STOVs has led to high-efficiency and ultra-compact systems for quantum optical applications [121], [164]. These examples suggest that the simple foundational models of topological phenomena will continue to play an important role for fundamental study and technological development as well.

Although substantial advances have been made thus far, extensive study is still ongoing in consideration of limitations and remaining challenges. The majority of topological GMR research has in many parts relied on phenomenological descriptions. For example, the polarization-vortex-charge approach provides a remarkably convenient classification and description scheme for BIC and unidirectional GMR formations. Nevertheless, it does not describe how the vortex charge itself emerges from the elementary scattering or diffraction processes for given structure geometry. Subsequently, applications of associated phenomena to practical device engineering are substantially limited in the absence of efficient design rules. Therefore, it is of great theoretical interest to quantitatively link elementary scattering amplitudes and structure geometry parameters to certain global properties which might lead to detailed design rules, discovery of new topological invariants, and development of novel optical devices.

In another consideration, previous study has been mostly limited to linear interaction regimes. A good starting point for the extension of the topic to the nonlinear interaction regime might be nonlinear parametric oscillations because they are described by 2 × 2 matrix-Hamiltonians which might be conveniently adapted to elementary topological Hamiltonians. The nonlinear extension is of particular importance not only for fundamental study but also for the development of active devices that take advantage of topological robustness and enhanced degrees of control freedom.
